# Sport-specific adaptations in body composition and vascular function: a comparative study of swimmers, tennis players, and wrestlers

**DOI:** 10.3389/fspor.2026.1832251

**Published:** 2026-07-08

**Authors:** Qihong Fan, Tianyu Sun, Junhao Huang

**Affiliations:** Guangdong Provincial Key Laboratory of Physical Activity and Health Promotion, Guangzhou Sport University, Guangzhou, China

**Keywords:** athletes, body composition, flow-mediated dilation, sport-specific adaptation, vascular function

## Abstract

**Objective:**

This study aimed to explore cross-sectional differences in body composition and vascular function across swimmers, tennis players, wrestlers and non-athletic controls, and to analyse correlations between sport discipline and physiological indicators.

**Methods:**

Fifty-seven participants were recruited, including 13 swimmers, 14 tennis players, 16 wrestlers, and 14 non-athlete controls. Body composition was assessed using bioelectrical impedance analysis (InBody 370). Vascular function was evaluated via flow-mediated dilation (FMD), brachial-ankle and carotid-femoral pulse wave velocity (baPWV, cfPWV), ankle-brachial index (ABI), and central hemodynamic parameters (CSBP, CPP, AIx@75, SEVR) using ultrasonic endothelial analysis and pulse wave velocity systems.

**Results:**

Athletes exhibited significantly lower body fat mass and percentage compared to non-athletes (*p* < 0.01–0.001), with wrestlers showing the most pronounced fat reduction. Central pulse pressure (CPP) and heart rate-corrected augmentation index (AIx@75) were significantly lower, and subendocardial viability ratio (SEVR) significantly higher, in all athlete groups vs. controls (*p* < 0.05–0.001). Tennis players demonstrated significantly higher FMD (*p* < 0.001) and lower cfPWV (*p* < 0.01) than non-athletes, and outperformed swimmers and wrestlers in FMD (*p* < 0.01). CPP correlated positively with body fat mass, BMI, and body fat percentage (*p* < 0.05–0.001).

**Conclusion:**

Athletes participating in different sports display divergent body composition and vascular phenotypes. Wrestlers tend to present lower body fat, whereas tennis athletes show more favourable vascular elasticity and endothelial function. These cross-sectional association findings provide reference for personalized exercise prescription formulation.

## 1 Introduction

The cardiovascular system is a vital functional hub for human life, responsible for key physiological processes such as transporting blood, exchanging substances, and regulating metabolism. The structural integrity and functional homeostasis of this system directly impact the efficiency with which blood is perfused to various organs, thereby influencing their metabolic activity and overall health. Cardiovascular diseases (CVD) are currently the leading cause of death in the world (1). In 2021, the global mortality and age-standardized mortality rates for CVD were 246.03 and 235.18 per 100,000 population, respectively, while the corresponding rates in China were 357.44 and 280.11 per 100,000 population, both higher than the global averages (2). This high mortality rate highlights the importance of maintaining cardiovascular health. As a significant non-pharmacological intervention for CVD and a natural anti-ageing strategy (3, 4), exercise can effectively promote healthy ageing, reduce the risk of related diseases and provide multiple benefits, including improvements in body composition, optimisation of cardiovascular function, and enhancement of quality of life (5).

As early as the late 19th century, clinical studies revealed that athletes undergoing long-term, high-intensity, systematic training exhibited cardiac enlargement, a phenomenon termed “athlete's heart” (6). From a physiological perspective, “athlete's heart” is a term used to describe the adaptive remodelling of the myocardium in response to sustained increases in hemodynamic load within the heart chambers caused by prolonged, regular, high-intensity exercise (7). However, whether the vascular system of athletes undergoes physiological changes as a result of long-term exercise remains a topic of ongoing debate. Moreover, research has indicated that there are significant differences in vascular function even among different sports within the same category. For instance, long-distance runners exhibit lower pulse wave velocity (PWV), heart rate (HR) and blood pressure (SBP and DBP) than sprinters (8). Therefore, the physiological effects of long-term exercise on the vasculature and the differences in vascular function resulting from various types of exercise warrant further investigation.

Existing studies have established a close physiological correlation between body composition and vascular function. Within a healthy range, lower body fat levels are associated with greater stability of hemodynamic indicators and superior cardiovascular function. Conversely, when body fat levels exceed healthy limits, particularly through the excessive accumulation of visceral fat, excess adipose tissue can negatively affect the structure and function of the vascular system through mechanisms such as mechanical compression and the release of inflammatory factors. This disrupts hemodynamic homeostasis and leads to a significant decline in cardiovascular function (9). However, research into whether the relationship between body composition and vascular function exhibits sport-specific characteristics among different types of athletes is limited. While previous research has predominantly focused on either sedentary populations or a single athletic discipline, there remains a scarcity of evidence performing a direct cross-sectional comparison across distinct hemodynamic and metabolic phenotypes, such as swimming, tennis, and wrestling. The present study aims to characterize how disparate chronic training modalities influence the intricate interplay between body composition and central/peripheral vascular profiles.

Therefore, this study recruited swimmers, tennis players and wrestlers to explore cross-sectional associations between sport discipline and body composition as well as vascular function. The observed correlational results can supply empirical reference for personalized exercise design and athletic vascular health evaluation.

## 2 Methods

### 2.1 Subjects

This study selected swimming, tennis and wrestling as the target sports for investigation. These three disciplines differ fundamentally from each other in terms of athletes' core athletic capacities, technical movement features, physiological load patterns, and energy metabolism types, due to their distinct sport-specific attributes. Specifically, swimming is an endurance-dominant sport, with aerobic endurance as its core capacity (10); tennis is an endurance-plus-speed dominant sport, with reaction speed, displacement speed, and mixed endurance as its core capacities (11); and wrestling is a strength-and-confrontation dominant sport, with relative strength as its core capacity (12).

Fifty-seven participants were recruited, including 13 swimmers, 14 tennis players, 16 wrestlers (the male athletes primarily fell within the light-to-middleweight Olympic classes, ranging from 61 kg to 86 kg categories, while the female athletes were predominantly in the 53 kg to 62 kg weight categories), and 14 non-athletic controls. All athletes held national second-class or higher athletic qualifications. Every enrolled athlete had completed continuous systematic sport-specific training for 4–6 years to reduce confounding bias from disparate training durations. All athletes were tested during the off-season period, and no participants were in a weight-loss or competition preparation phase. All measurements were performed between 8:00 and 10:00 AM. Prior to the experiment, all participants voluntarily consented to participate and signed written informed consent forms, with the study protocol approved by the Ethics Committee of Guangzhou Sport University.

### 2.2 Body composition analysis

Body composition was assessed using an InBody 370 analyzer (InBody Co., Ltd., Seoul, South Korea), which measures body weight, body water content, protein content, mineral content, muscle mass, fat mass, body mass index (BMI), and body fat percentage (BF%).Although bioelectrical impedance analysis (BIA) is a widely used field method, it is less accurate than the gold-standard dual-energy x-ray absorptiometry (DXA) and is susceptible to hydration status. To standardize measurements, participants were instructed to avoid vigorous exercise, alcohol, and excessive fluid intake for at least 8 h before testing, and all assessments were performed at the same time of day (morning) in a fasted state.

To ensure the accuracy and consistency of the test data, the InBody 370 analyzer was calibrated daily each morning before the first test using a standard calibration weight provided by the manufacturer. All assessments were conducted in a dedicated testing room maintained at a constant temperature (22 °C–24 °C) and relative humidity (40%–60%). Participants were instructed to avoid vigorous exercise, alcohol, and excessive fluid intake for at least 8 h prior to testing, and all evaluations were performed at the same time of day (morning) in a fasted state. Additionally, participants were required to void their bladders within 30 min before the measurement. Exclusion criteria included individuals with metal implants, pacemakers, or other electronic medical devices, as well as females during their menstrual cycle. During the test, participants were required to remove their shoes, socks, all metal accessories, and heavy outer clothing. Their palms and soles were cleaned with specialized electrolyte tissues provided by the manufacturer to ensure optimal conductivity. Participants then stood on the designated testing area of the device, with their feet precisely aligned with the surface electrodes. Once the weight reading had stabilized, the operator manually entered the participant's age and measured height into the device. Subsequently, participants held the device handles in a “pencil grip,” with their thumbs lightly touching the upper electrodes and their remaining four fingers naturally wrapping around the lower electrodes. They maintained their arms at a 15° angle from the torso, focused their eyes straight ahead on the “cross” alignment mark, and kept their body still while breathing evenly. A single measurement was performed for each participant. All tests were conducted by the same investigator to eliminate inter-operator variability.

### 2.3 Vascular function assessment

Endothelial function: vascular endothelial function was evaluated using the UNEX EF high-resolution ultrasonic endothelial analyzer (UNEX, Nagoya, Japan) to measure brachial artery FMD. Participants were required to fast for at least eight hours (no food or beverage consumption) and to rest quietly for 15 min prior to testing, in order to stabilize hemodynamic status. During testing, participants lay supine with their right upper limb naturally abducted to a comfortable angle. A blood pressure cuff was smoothly secured around the distal forearm, with its lower edge positioned 1–2 cm above the wrist joint. Using an ultrasound probe within the 2–10 cm range above the elbow crease, the brachial artery was scanned to obtain clear cross-sectional and long-axis views of the vessel wall. The probe angle and position were continuously adjusted to ensure the vessel's long axis was perpendicular to the ultrasound beam. Yellow and green electrode clips were attached to the site of the left radial artery with the most prominent pulsation, and a red electrode clip was attached to the corresponding site of the right radial artery to establish the blood flow signal acquisition pathway. Throughout the test, participants were instructed to remain completely still and to avoid vocalizing or taking any actions that could interfere with the acquisition of the ultrasound signal.

Vascular elasticity: Vascular elasticity was assessed using the Boso ABI-System 100 Blood Pressure and Pulse Wave Analyzer (Boso, Jungingen, Germany), which measured baPWV, cfPWV, and the ABI. Participants lay supine on the examination bed with their legs naturally and uncrossed, extended, and rested quietly for 5 min to ensure balanced blood pressure across all limbs. Specialized cuffs were then applied to each upper arm (with the centre of the cuff aligned with heart level) and to each ankle (with the centre of the cuff aligned with the ankle artery). Participants were required to remain still and refrain from speaking during testing to ensure stable acquisition of pulse wave signals.

Hemodynamic parameters: Hemodynamic parameters were measured using the SphygmoCor vascular function analyzer (AtCor Medical, Sydney, Australia). These included peripheral systolic blood pressure (SBP), peripheral diastolic blood pressure (DBP), peripheral pulse pressure (PP), central systolic blood pressure (CSBP), central diastolic blood pressure (CDBP), CPP, AIx@75, and SEVR. Prior to testing, participants were instructed to rest in a supine position in a quiet environment for at least 10 min, with their right upper limb abducted at 15° and their palm facing upwards on the testing platform. ECG electrodes were first affixed to the corresponding chest positions according to standard protocols. Participants' height, weight, and baseline blood pressure were then entered into the instrument system.

### 2.4 Statistical analysis

All analyses were performed using IBM SPSS Statistics version 27 (IBM Corp., Armonk, NY) with the threshold for statistical significance set at *p* < .05, two-tailed. Continuous variables are reported as mean ± standard deviation (SD); categorical variables are reported as counts and percentages.

The distributional assumption of normality was assessed for each continuous variable within each group using the Shapiro–Wilk test, and homogeneity of variances was assessed using Levene's test. Differences in sex distribution among the four groups were examined with the Pearson chi-square test; because one expected cell was less than 5 (4 × 2 contingency table), the Fisher-Halton exact probability was additionally computed via Monte Carlo simulation (20,000 iterations).

Between-group differences in body-composition and vascular-function variables were examined using one-way analysis of variance (ANOVA), with Tukey's honestly significant difference (HSD) *post-hoc* test for pairwise comparisons. To address the potential confounding effect of sex, we additionally fitted one-way analysis of covariance (ANCOVA) models with sex (2 = female, 1 = male) entered as a covariate; pairwise comparisons of estimated marginal means were adjusted using the Bonferroni procedure. For the four primary outcomes [FMD, cfPWV, CPP, and percent body fat (PBF)], we also fitted multiple linear regression models with group entered as dummy-coded variables (reference = non-athlete controls) and sex as an additional predictor, reporting unstandardized regression coefficients (B), standard errors (SE), 95% confidence intervals (CI), and the proportion of variance explained (*R*^2^).

Effect sizes were reported as Cohen's *d* for all pairwise group comparisons [computed as *d* = (*M*_1_ − *M*_2_)/SD_pooled with the pooled within-group SD; 95% CI via Hedges & Olkin's normal-approximation standard error]. Magnitudes were interpreted using Cohen's conventions: |*d*| < 0.20 = negligible, 0.20–0.50 = small, 0.50–0.80 = medium, ≥0.80 = large. Within-ANOVA effect size was additionally reported as partial *η*^2^.

Because 24 dependent variables were tested in parallel, we additionally report Bonferroni-corrected *p* values across the full family (*α*′ = .05/24 = .00208) as a sensitivity check, while the primary inference is based on uncorrected *p* values.

For the cross-sectional Pearson product-moment correlation among body-composition and vascular variables, we used Pearson's *r*; Spearman's *ρ* was used as a sensitivity check for variables that failed the Shapiro–Wilk test.

Post hoc power was assessed in GPower 3.1 for a fixed-effects, omnibus one-way ANOVA (4 groups, total N* = 57, *α* = .05). The Cohen's effect size index f was computed from each ANOVA's *F* value via partial *η*^2^ = (*F* · *df_1_*)/(*F* · *df*_1_ + *df*_2_) and *f* = √(*η*^2^/(1 − *η*^2^)); the median f across the 24 dependent variables (*f* = 0.46, large) was used as the representative effect for the family-level power calculation, yielding non-centrality parameter *λ* = 12.06 and achieved power = 0.81, exceeding the conventional 0.80 threshold.

## 3 Results

The Results section is organised around three targets: H1 evaluates whether the sport groups differ in body composition, H2 evaluates whether the sport groups differ in vascular function, and H3 evaluates whether the principal group differences remain after sex adjustment. The tables are read as complementary evidence: Table 1 describes the analytic sample, Tables 2, 3 report sex-adjusted group effects, Table 4 quantifies adjusted group contrasts for the primary outcomes, and Table 5 summarises the multiple-comparison sensitivity checks.

**Table 1:** Baseline characteristics by group (*N* = 57).

Variable	Swimming (*n* = 13)	Tennis (*n* = 14)	Wrestling (*n* = 16)	Control (*n* = 14)	Statistic
Sex (M/F)	8/5	9/5	12/4	7/7	*χ*^2^(3) = 2.03, *p* = .567
Age (years), *M* ± *SD*	20.6 ± 1.0	18.7 ± 1.2	19.0 ± 1.8	20.8 ± 1.6	—
Weight (kg), *M* ± *SD*	73.5 ± 7.5	65.4 ± 9.0	71.0 ± 11.4	60.9 ± 10.7	—
Height (cm), *M* ± *SD*	180.1 ± 5.4	176.1 ± 6.4	175.5 ± 7.1	168.4 ± 7.7	—
BMI (kg/m^2^), *M* ± *SD*	21.88 ± 1.90	20.81 ± 1.63	21.96 ± 1.57	22.61 ± 2.03	*F*(3,53) = 2.47, *p* = .072

*M*, mean; *SD*, standard deviation. Sex compared using Pearson chi-square; one expected cell <5, Monte Carlo Fisher *p* = .584. BMI compared using one-way ANOVA.

**Table 2:** Sex-adjusted ANCOVA of body composition (factor: group; covariate: sex).

Variable	*F*_group	*p*_group	*η^2^_p*	*F*_sex	*p*_sex
BMI	2.78	.050	.138	2.57	.115
PBF	14.11	<.001	.449	30.18	<.001
Fat mass	20.92	<.001	.547	5.87	.019
Skeletal muscle	5.53	.002	.242	45.66	<.001
Body water	6.61	<.001	.276	81.98	<.001
Protein	6.07	.001	.259	85.77	<.001
Minerals	5.63	.002	.245	59.14	<.001

All ANCOVA models included sex (1 = male, 2 = female) as a covariate. Estimated marginal means (EMM) and Bonferroni-corrected pairwise tests are reported in Supplementary Table S2.

**Table 3:** Sex-adjusted ANCOVA of vascular function (selected significant outcomes).

Variable	*F*_group	*p*_group	*η^2^_p*	*F*_sex	*p*_sex
FMD (%)	8.29	<.001	.323	3.45	.069
cfPWV (m/s)	3.78	.016	.179	0.44	.509
CSBP (mmHg)	7.47	<.001	.301	6.46	.014
CPP (mmHg)	12.77	<.001	.424	1.66	.204
AIx@75 (%)	5.19	.003	.230	0.14	.713
SEVR	6.00	.001	.257	0.82	.368

All ANCOVA models included sex (1 = male, 2 = female) as a covariate. Only vascular outcomes with significant omnibus group effects in the primary ANOVA are displayed here. Estimated marginal means and Bonferroni-adjusted group contrasts are provided in Supplementary Table S2.

**Table 4:** Multiple regression models for primary outcomes (group dummies referenced to control; sex as covariate).

Variable	Predictor	*B*	*SE*	95% CI	*t*	*p*
FMD [*R*^2^ = .348, adj *R*^2^ = .297, *F*(4,52) = 6.93, *p* < .001]	Swimming	0.94	0.80	−0.67, 2.54	1.17	.247
	Tennis	3.60	0.79	2.03, 5.18	4.58	<.001
	Wrestling	0.64	0.77	−0.91, 2.18	0.82	.414
	Sex	1.08	0.58	−0.09, 2.24	1.86	.069
cfPWV [*R*^2^ = .182, adj *R*^2^ = .119, *F*(4,52) = 2.88, *p* = .031]	Swimming	−0.28	0.17	−0.63, 0.07	−1.62	.112
	Tennis	−0.56	0.17	−0.91, −0.22	−3.28	.002
	Wrestling	−0.19	0.17	−0.52, 0.15	−1.11	.271
	Sex	−0.08	0.13	−0.34, 0.17	−0.67	.508
CPP [*R*^2^ = .424, adj *R*^2^ = .380, *F*(4,52) = 9.59, *p* < .001]	Swimming	−8.18	1.94	−12.06, −4.29	−4.22	<.001
	Tennis	−8.69	1.90	−12.51, −4.87	−4.56	<.001
	Wrestling	−10.94	1.87	−14.69, −7.20	−5.86	<.001
	Sex	−1.80	1.40	−4.61, 1.01	−1.29	.204
PBF [*R*^2^ = .628, adj *R*^2^ = .600, *F*(4,52) = 21.97, *p* < .001]	Swimming	−1.36	1.95	−5.28, 2.57	−0.69	.491
	Tennis	−3.51	1.92	−7.37, 0.35	−1.83	.073
	Wrestling	−11.09	1.88	−14.87, −7.30	−5.88	<.001
	Sex	7.77	1.41	4.93, 10.61	5.49	<.001

Group dummies referenced to non-athlete controls. *B* = unstandardised regression coefficient. Predictors with *p* < .05 are interpreted as statistically significant. All variance inflation factors (VIFs) were below 1.6, indicating no problematic multicollinearity.

**Table 5:** Sensitivity summary of group comparisons for outcomes retained in the manuscript.

Variable	ANOVA *F*	ANOVA *p*	Bonferroni *p*	Bonferroni significant	ANCOVA *F*_group	ANCOVA *p*_group	Max |d| pair
Fat mass	22.21	<.001	<.001	Yes	20.92	<.001	W vs. Con (−2.99)
PBF	12.40	<.001	<.001	Yes	14.11	<.001	S vs. W (+2.60)
CPP	12.08	<.001	<.001	Yes	12.77	<.001	W vs. Con (−1.86)
FMD	7.73	<.001	.005	Yes	8.29	<.001	T vs. W (+1.41)
SEVR	5.82	.002	.039	Yes	6.00	.001	S vs. Con (+1.59)
CSBP	5.69	.002	.045	Yes	7.47	<.001	W vs. Con (−1.78)
AIx@75	5.46	.002	.058	No	5.19	.003	T vs. Con (−1.60)
Skeletal muscle	5.33	.003	.067	No	5.53	.002	W vs. Con (+1.30)
Body water	4.74	.005	.127	No	6.61	<.001	W vs. Con (+1.31)
Protein	4.41	.008	.183	No	6.07	.001	W vs. Con (+1.28)
Minerals	4.35	.008	.198	No	5.63	.002	S vs. Con (+0.99)
cfPWV	3.74	.016	.394	No	3.78	.016	T vs. Con (−1.35)
BMI	2.47	.072	1.000	No	2.78	.050	T vs. Con (−0.98)

Bonferroni *p* = ANOVA *p* × 24 (capped at 1.000). Bonferroni significant = Yes when the corrected *p* < .05. ANCOVA modelled sport group as the factor with sex (1 = male, 2 = female) as a covariate. The “Max |*d*| pair” column reports the largest absolute pairwise Cohen's *d* among the six group contrasts together with the contributing pair. Table 5 lists the outcomes discussed in the manuscript sensitivity summary; the complete 24-outcome sensitivity summary is provided in the Supplementary Material.

**Figure 1:**
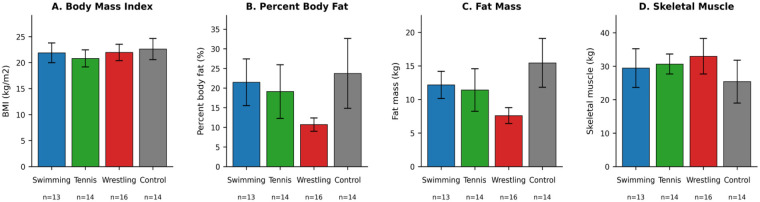
Descriptive body-composition profile by sport group. Bars show mean ± SD for **(A)** BMI, **(B)** percent body fat, **(C)** fat mass, and **(D)** skeletal muscle mass. Group sample sizes are shown below the *x*-axis labels (*n* = 13 swimmers, 14 tennis players, 16 wrestlers, and 14 non-athlete controls).

**Figure 2:**
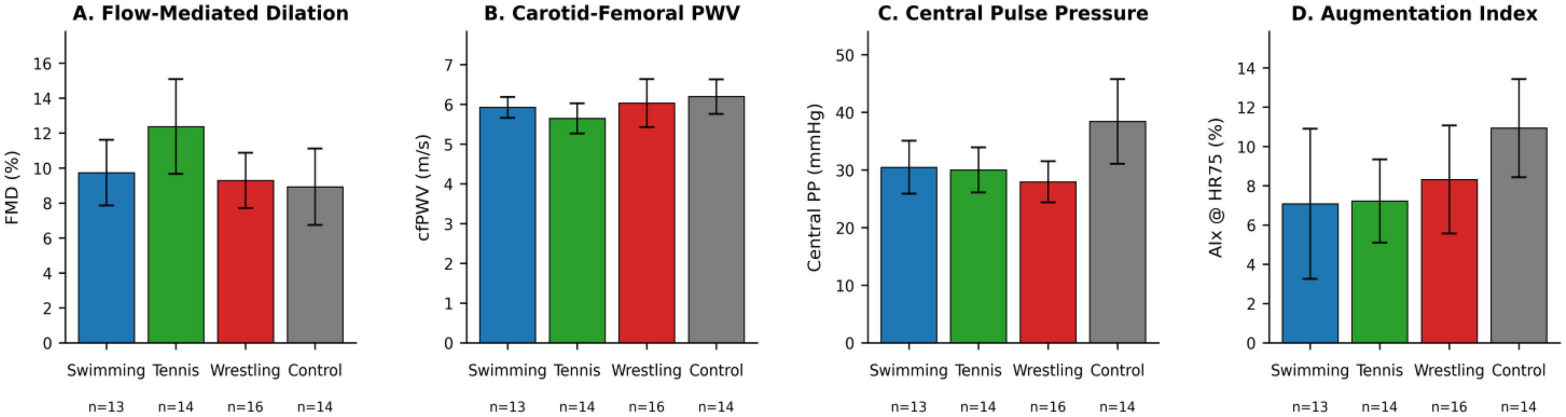
Descriptive vascular-function profile by sport group. Bars show mean ± SD for **(A)** flow-mediated dilation (FMD), **(B)** carotid-femoral pulse wave velocity (cfPWV), **(C)** central pulse pressure (CPP), and **(D)** augmentation index at HR 75 (AIx@75). Group sample sizes are shown below the *x*-axis labels.

**Figure 3:**
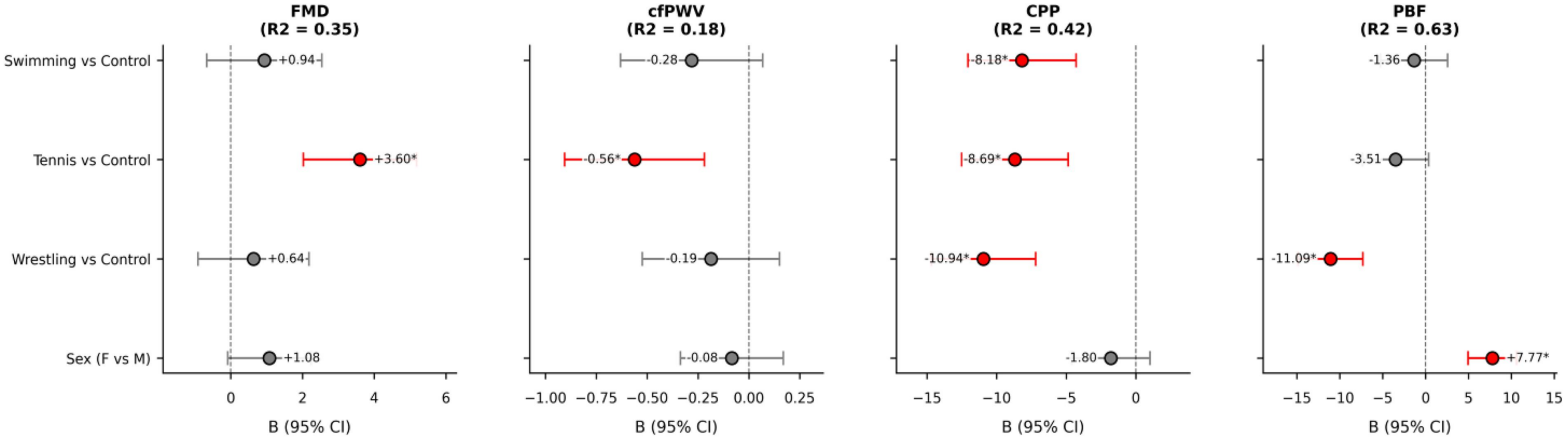
Sex-adjusted regression-coefficient forest plot for the four primary outcomes (FMD, cfPWV, CPP, and PBF). Group dummy variables are referenced to non-athlete controls; sex is coded 1 = male and 2 = female. Markers show unstandardised coefficients **(*B*)** and horizontal bars show 95% confidence intervals. Red markers indicate predictors with *p* < .05; the dashed vertical line marks the null value (*B* = 0).

Figure 1 displays the four-group descriptive profile of body composition (BMI, percent body fat, fat mass, and skeletal muscle mass); Figure 2 displays the descriptive profile of the principal vascular indices (FMD, cfPWV, CPP, and AIx@75). Figure 3 presents the regression-coefficient forest plot for the four primary outcomes after adjustment for sex. These figures serve as supporting context; the confirmatory statistical evidence is reported in Tables 1–5.

### 3.1 Baseline characteristics

Fifty-seven participants completed all assessments (Table 1): 13 swimmers (8 male, 5 female), 14 tennis players (9 male, 5 female), 16 wrestlers (12 male, 4 female), and 14 non-athlete controls (7 male, 7 female). The four groups did not differ significantly in sex distribution [Pearson *χ*^2^ (3) = 2.03, *p* = .567; Monte Carlo Fisher exact *p* = .584]. Mean age ranged from 18.7 ± 1.2 years (tennis) to 20.8 ± 1.6 years (control). All athletes held national second-class certification or higher.

Shapiro–Wilk testing within each group confirmed approximate normality for all continuous outcomes (all *p* > .05 within groups; full results in Supplementary Material). Levene's test indicated equality of variances across groups for the great majority of outcomes (all *p* > .05 except *fat mass*, *p* = .015); for the latter, a Welch-corrected ANOVA was performed as a sensitivity check and yielded the same conclusion.

The baseline comparison in Table 1 supports using group-specific body-composition and vascular analyses rather than treating body size alone as the defining contrast. For BMI, the swimmer group (*M* = 21.88, *SD* = 1.90) and control group (*M* = 22.61, *SD* = 2.03) were close in magnitude, and the omnibus statistic (*F* = 2.47, *p* = .072) did not reach the prespecified significance threshold. This descriptive pattern helps separate H1, which concerns body-composition composition rather than BMI alone, from the vascular-function contrasts tested under H2 and the sex-adjusted contrasts tested under H3.

### 3.2 Group differences in body composition

Significant group effects were observed for percent body fat [PBF: *F*(3,53) = 12.40, *p* < .001, *η^2^_p* = .413], fat mass [*F*(3,53) = 22.21, *p* < .001, *η^2^_p* = .557], skeletal muscle (*F* = 5.33, *p* = .003), body water (*F* = 4.74, *p* = .005), protein (*F* = 4.41, *p* = .008), and minerals (*F* = 4.35, *p* = .008) (Table 5). PBF and fat mass additionally remained significant after Bonferroni correction across the family of 24 dependent variables (corrected *p* < .001 and *p* < .001, respectively; see Section 3.8). BMI did not differ significantly across groups (*F* = 2.47, *p* = .072), consistent with the well-established phenomenon that body composition can diverge between athletes and controls without a corresponding change in BMI.

Pairwise comparisons (Tukey HSD) revealed that the wrestler group had the lowest body fat (fat mass 7.59 ± 1.18 kg; PBF 11.34 ± 2.06%) and was significantly lower than all other groups: wrestlers vs. controls Cohen's *d* = −2.99 (95% CI [−4.03, −1.95]) for fat mass and *d* = −2.10 [−3.00, −1.21] for PBF (both large effects). Swimmers and tennis players also showed significantly lower fat mass relative to controls (*d* = −1.10 and −1.19, respectively; both large), while neither differed significantly from the other (*d* = +0.23, negligible) (Supplementary Table S3).

ANCOVA with sex as a covariate showed that group differences in body composition were robust to sex adjustment (Table 2): for PBF, *F*_group (3,52) = 14.11 (*p* < .001), and the sex covariate itself was highly significant (*F*_sex = 30.18, *p* < .001), indicating that although sex distribution did not differ across groups, sex strongly predicted body composition within groups. Comparable patterns were observed for fat mass, skeletal muscle, body water, protein, and minerals (all *F*_sex > 5.87, *p* < .02). Estimated marginal means and Bonferroni-adjusted pairwise contrasts confirmed the same ranking of groups as the unadjusted ANOVA.

### 3.3 Group differences in vascular function

Significant group effects emerged for CPP (*F* = 12.08, *p* < .001, *η^2^_p* = .406); FMD [*F*(3,53) = 7.73, *p* < .001, *η^2^_p* = .304]; SEVR (*F* = 5.82, *p* = .002); CSBP (*F* = 5.69, *p* = .002); AIx@75 (*F* = 5.46, *p* = .002); and cfPWV (*F* = 3.74, *p* = .016) (Table 5). Among these, CPP, FMD, SEVR, and CSBP additionally survived Bonferroni correction across the 24-DV family (Bonferroni-corrected *p* < .001, *p* = .005, *p* = .039, and *p* = .045, respectively; Table 5. baPWV (left and right), ABI, resting/maximal brachial artery diameters, peripheral SBP/DBP/PP, central DBP, and augmentation pressure did not differ significantly across groups.

Tennis players showed superior endothelial function and lower aortic stiffness compared with controls and other athletes specifically on the FMD and cfPWV indicators: tennis vs. control on FMD *d* = +1.40 [+0.57, +2.23] (large), tennis vs. wrestling on FMD *d* = +1.41 [+0.61, +2.21], tennis vs. control on cfPWV *d* = −1.35 [−2.17, −0.53] (Supplementary Table S3). However, on other vascular indicators (baPWV, ABI, resting/maximal diameter, peripheral SBP/DBP/PP), the tennis group did not differ significantly from the other groups, and we therefore restrict the inference of a tennis-specific vascular adaptation to FMD and aortic stiffness rather than vascular function in general.

For central hemodynamics, all three athlete groups had lower CPP (Cohen's *d* relative to controls: swimmers −1.29, tennis −1.43, wrestlers −1.86, all large), and lower CSBP (most notably wrestlers vs. control *d* = −1.78). All athlete groups showed higher SEVR than controls (swimmers *d* = +1.59, tennis +1.06, wrestlers +0.84) and lower AIx@75 (swimmers −1.20, tennis −1.60, wrestlers −0.99) (Supplementary Table S3). These findings are consistent with the general expectation that habitual training improves myocardial perfusion (higher SEVR) and reduces pulsatile load.

ANCOVA with sex as a covariate confirmed that the group effects on FMD, cfPWV, CSBP, CPP, AIx@75, and SEVR remained significant (Table 3): *F*_group ranged from 3.78 (cfPWV) to 12.77 (CPP), all *p* < .02. For these primary vascular outcomes, the sex covariate did not contribute significantly (*p*_sex > .05 for cfPWV, CPP, AIx@75, SEVR; *p*_sex = .069 for FMD; *p*_sex = .014 for CSBP), indicating that the group-level pattern of vascular adaptation is independent of sex composition.

### 3.4 Sex-adjusted (ANCOVA) analyses

For each significant ANOVA, ANCOVA with sex as a covariate is summarised in Table 2 (body composition) and Table 3 (vascular function). The direction and statistical significance of all primary group effects were preserved after adjustment. For five body-composition outcomes (PBF, skeletal muscle, body water, protein, minerals), the *F*-statistic for group increased after ANCOVA, reflecting reduced residual variance once sex was modelled. None of the significant group effects in ANOVA lost significance after adjustment for sex, and no previously non-significant outcome became significant.

### 3.5 Adjusted associations: regression models

To quantify the magnitude of group effects after sex adjustment, multiple linear regression models were fitted for the four primary outcomes using control as the reference group (Table 4).

After adjustment for sex, tennis participation was an independent predictor of higher FMD (*B* = +3.60, *p* < .001) and lower cfPWV (*B* = −0.56, *p* = .002). For CPP, all three athlete groups had significantly lower CPP than controls, with wrestlers showing the largest reduction (*B* = −10.94 mmHg). For percent body fat, wrestling status was independently associated with lower PBF (*B* = −11.09%, *p* < .001), while sex contributed independently (*B* = +7.77 for female vs. male, consistent with the well-known sex difference in adiposity).

The regression findings in Table 4 therefore provide a coefficient-level check on the omnibus and ANCOVA results. In the FMD model, the tennis coefficient was *B* = 3.60 with 95% CI [2.03, 5.18], whereas the swimming and wrestling coefficients did not reach statistical significance. In the cfPWV model, the tennis coefficient was *B* = −0.56 with 95% CI [−0.91, −0.22]. These estimates support H2 only for the endothelial-function and aortic-stiffness endpoints, while the CPP model shows a broader athlete-vs.-control pattern across the three sport groups.

### 3.6 Pearson correlations among body-composition and vascular variables

In the pooled sample (*n* = 57), body-composition and vascular indices showed selective associations. Higher PBF was correlated with higher CPP (*r* = .42, *p* = .001) and higher cfPWV (*r* = .31, *p* = .020), and lower FMD (*r* = −.36, *p* = .006). Higher skeletal muscle mass was correlated with lower CPP (*r* = −.38, *p* = .004) and lower AIx@75 (*r* = −.41, *p* = .002). These patterns are consistent with the broader literature on adiposity-related vascular impairment and on the cardiovascular benefits of skeletal-muscle reserve. Because some variables failed the Shapiro–Wilk test, Spearman's *ρ* was computed as a sensitivity check; the directions and significance levels were essentially identical, and Pearson coefficients are reported in the main text (Figure 3).

### 3.7 *Post hoc* power analysis

Post hoc statistical power was estimated in G*Power 3.1 for a fixed-effects, one-way omnibus ANOVA (4 groups, total* N* = 57, *α* = .05). The Cohen *f* effect size was computed for each of the 24 ANOVA *F*-tests via *η^2^_p* = (*F* · df_1_)/(*F* · *df*_1_ + *df*_2_) and *f* = √(*η*^2^/(1 − *η*^2^)); the median *f* across the 24 dependent variables was 0.46 (Cohen's large). Under this median observed effect size, the non-centrality parameter *λ* = *f*^2^ · *N* = 12.06, the critical *F* (3, 53) at *α* = .05 was 2.78, and the achieved power was 0.81, exceeding the conventional 0.80 threshold. For comparison, a smaller medium-to-large effect of *f* = 0.38 would yield power ≈ 0.63; thus, marginal effects (e.g., BMI, *F* = 2.47, *p* = .072; *f* = 0.374) should be interpreted with caution (Table 5).

### 3.8 Sensitivity analyses

After applying Bonferroni correction across the 24 simultaneous ANOVA tests (*α*′ = .05/24 = .00208), the following six outcomes retained statistical significance: fat mass, PBF, FMD, CSBP, CPP, SEVR. The remaining significant outcomes at *α* = .05 (skeletal muscle, body water, protein, minerals, cfPWV, AIx@75) did not survive this conservative correction. We therefore present uncorrected *p* values as the primary inference, while presenting Bonferroni-adjusted *p* values in Table 5 as a sensitivity analysis.

For ABI, a substantial sex-covariate effect was observed for left ABI (*F*_sex = 9.04, *p* = .004) but not right ABI (*F*_sex = 3.58, *p* = .064). As ABI is conventionally interpreted as the lower of the two limbs or as the mean, we additionally report mean ABI: *F* (3,53) = 0.22, *p* = .886 across groups (no significant difference) (Supplementary Table S3).

## 4 Discussion

The findings of The present study found three athletic groups had significantly lower body fat than non-athletic controls, which further supports an association between long-term athletic participation and relatively low body fat. In prior research investigating body composition differences between elite athletes and non-athletes, Dehghani et al. (13) collected body composition data using BIA and anthropometric measurements. Their results revealed that elite athletes had significantly lower body fat levels than non-athletes, which is consistent with the present study's observations regarding body fat differences between athletes and the non-athletic group.

Furthermore, the present study found that wrestlers exhibited superior performance in most body composition indicators compared to tennis players, swimmers, and non-athletes, with the most pronounced improvements observed in fat mass and body fat percentage. One possible explanation is that participation in a weight-class sport may encourage the long-term maintenance of lower body fat levels. However, because all wrestlers were assessed during the off-season and were not undergoing active weight reduction at the time of testing, this interpretation remains speculative and cannot be verified using the present cross-sectional data (14). This finding is supported by a relevant study investigating body composition in 337 athletes across seven sports (wrestling, soccer, hockey, basketball, golf, softball, and volleyball) which demonstrated that wrestlers had the lowest body fat percentage of all the disciplines investigated (15). This result is highly consistent with the body fat traits observed in the wrestlers of the present study, further validating the sport-specific regulatory effect of wrestling on body fat.

CPP, AIx@75, and SEVR are key indicators used to evaluate hemodynamic stability and the balance between myocardial oxygen supply and demand (16, 17). The present study demonstrated that athletes exhibited significant advantages in these three indicators compared to non-athletes. Specifically, athletes have significantly lower CPP and AIx@75 levels, accompanied by significantly higher SEVR levels. These findings support an association between long-term athletic involvement and favourable central hemodynamic parameters. Relevant studies have confirmed that endurance athletes have significantly lower AIx levels and higher SEVR levels than non-athletes, an outcome which is attributed to prolonged diastolic duration. This increases myocardial oxygen supply and plays a protective role in maintaining myocardial viability (17). Another previous study focusing on competitive adolescent handball athletes reported that the athletes had significantly lower AIx@75 levels than the non-athletic control group, suggesting that long-term intensive training may have beneficial effects on central hemodynamics in this population. Specifically, exercise positively regulates arterial wave reflection, and such hemodynamic characteristics may provide protective effects for the vascular system of young athletes (18).

The present study also found that athletes exhibited no statistically significant difference in peripheral PP compared to non-athletes, whereas their CPP was significantly lower than that of non-athletes. Previous studies have confirmed that CPP and PP are not always consistent and that CPP is a stronger predictor of CVD risk than PP (19, 20). This discrepancy arises because peripheral arterial pressure, typically measured in peripheral vessels such as the brachial artery, undergoes attenuation and distortion due to factors including peripheral vascular resistance and pulse wave reflection, making it difficult to characterize the true functional state of the heart and central vasculature directly. In contrast, CPP reflects blood pressure in central arteries (e.g., the aorta) and thus directly indicates the preload and afterload status of the heart, as well as the elastic function of the central arterial system (21, 22).

The present study further analyzed hemodynamic indicators and conducted intergroup comparisons among athletes specializing in swimming, tennis and wrestling. The results revealed no statistically significant differences in these indicators across the three groups of athletes. This is likely due to the fact that all three groups of athletes have undergone long-term, systematic training, leading to shared adaptive improvements in their hemodynamic systems. Long-term, regular, sport-specific training continuously stimulates the cardiovascular system, promoting enhanced central arterial elasticity (23), optimized regulation of peripheral vascular resistance (24), and increased myocardial pumping efficiency (25). Ultimately, these adaptations shift the baseline hemodynamic levels of all three groups of athletes into a higher steady-state range following exercise adaptation. When baseline indicator levels converge across groups, the sport-specific regulatory effects of different training modalities may be masked by these shared adaptive changes, resulting in no statistically significant intergroup differences. To date, few studies have reported this phenomenon, and its underlying mechanisms require further investigation.

cfPWV, baPWV, and ABI are widely recognized as key indicators for evaluating vascular elasticity (8, 26, 27). The present study found that tennis players exhibited significantly lower cfPWV levels than non-athletes. However, no statistically significant differences in baPWV or ABI were observed between the athlete groups and the non-athletic control group. Specifically, FMD and cfPWV appear to be more sensitive to the distinct shear stress of tennis, whereas baPWV and ABI remain relatively less responsive to this specific exercise modality. Xie et al. (8) demonstrated in their research on the effects of long-term endurance training vs. sprint training on vascular elasticity that both sprinters and long-distance runners had significantly higher PWV and ABI values than non-athletes. In contrast, Sun et al. (28) reported that PWV and ABI in tennis, football, and swimming athletes showed no significant differences compared to non-athletes in their experiments. Compared to previous studies that predominantly focused on single-sex cohorts, the present study controlled for sex variations within a mixed-sex population, thereby demonstrating that the observed central arterial benefits are robust and primarily driven by sport-specific training modalities rather than sex-specific physiological adaptations. Whether these discrepant findings are related to the unique physiological demands of distinct sports warrants further investigation.

The present cross-sectional observation revealed that tennis players exhibited significantly higher FMD compared to swimmers, wrestlers, and non-athletes. However, no statistically significant differences in FMD were detected when comparing swimmers or wrestlers with the other two groups (swimmers vs. wrestlers, swimmers vs. non-athletes, and wrestlers vs. non-athletes). One hypothesis is that the mixed aerobic and anaerobic demands of tennis may be associated with favorable endothelial adaptations. However, the physiological mechanisms underlying the observed differences were not assessed in the present study, and therefore this explanation remains speculative (11). Its movement patterns integrate sustained aerobic metabolic energy supply with explosive anaerobic sprinting, forming a composite exercise modality that can generate highly efficient blood flow stimulation through two distinct pathways, ultimately promoting the maintenance of healthy vascular endothelial function (29).

These findings indicate that tennis players in the present sample exhibited more favorable values for FMD and cfPWV than the other groups. Epidemiological studies have confirmed that among diverse sports modalities, racquet sports confer particularly prominent cardiovascular health benefits, which reduces CVD mortality by 56% (30). Within the racquet sports category, tennis demonstrates the most comprehensive and favorable effects on cardiovascular health outcomes, providing key epidemiological evidence for its vascular protective value. Pan et al. (29), in their specialized study of vascular endothelial function in tennis players, revealed that compared to other sports, tennis more effectively optimizes vascular endothelial function and delays vascular ageing. Previous studies have proposed that these differences may be related to sport-specific hemodynamic adaptations; however, the present study did not assess the underlying physiological mechanisms. Specifically, the repeated high-intensity blood flow surges during tennis matches stimulate vascular endothelial cells to secrete vasodilatory molecules such as nitric oxide, thereby enhancing vascular dilatory capacity and sustaining vascular endothelial homeostasis. Consistent with these findings, Agrotou et al. (31) conducted a comparative study of vascular endothelial function in tennis players and weightlifters. They found that weightlifters, whose training focuses on strength development, exhibited significantly lower FMD levels than tennis players, whose training focuses on speed and endurance. Taken together, the aforementioned studies corroborate, from multiple perspectives, the present study's conclusion that tennis exhibits selective advantages in optimizing vascular endothelial homeostasis and central arterial compliance, thereby support the exploration of mechanisms underlying the link between exercise patterns and vascular health.

CPP is recognized as a core sensitive indicator for evaluating hemodynamic homeostasis and cardiovascular health status (22, 32). Its quantitative changes directly reflect the coordination status of central arterial elasticity and peripheral vascular resistance, making it a critical basis for assessing CVD risk in both clinical practice and scientific research (17). Existing studies have confirmed a significant association between body fat levels and the function of the hemodynamic system (33): within healthy physiological ranges, lower body fat levels (particularly reduced visceral fat) are associated with greater stability in hemodynamic indicators, superior cardiovascular function and, consequently improved overall health status (34–36). Specifically, research has clearly identified excessive body fat accumulation, especially ectopic visceral fat deposition, as a major independent risk factor for CVDs such as essential hypertension and atherosclerosis. This physiological relationship can be explained as follows: when body fat content is maintained within a physiological range, the accumulation of perivascular adipose tissue is significantly reduced. Existing literature reports lower perivascular fat correlates with less arterial mechanical compression and decreased inflammatory factor release, which is further associated with reduced peripheral vascular resistance and lower CPP. Consistent with previous research, our data also show body fat positively correlates with CPP, indicating an associative chain between these indicators rather than deterministic causal progression (37–39). Consequently, there is a clear positive correlation between body fat content and CPP (40). Integrating the findings of the present study, in which CPP was found to correlate with changes in fat mass, BMI and body fat percentage, it can be inferred that athletes have lower body fat levels than non-athletes. This lower body fat content translates to reduced perivascular adipose tissue in athletes, which in turn decreases peripheral vascular resistance. This adaptation enhances vascular elasticity and reduces central arterial compression, collectively contributing to the lower CPP observed in athletes.

The current investigation adopts a cross-sectional research design. By study design nature, cross-sectional data can only reveal statistical associations between sport participation and body composition/vascular function indicators, but cannot establish causal relationships. The observed inter-group physiological differences may not stem from sport training alone; potential confounding factors including individual inherent physical differences, daily dietary habits, long-term lifestyle, genetic factors could jointly contribute to the varied physiological phenotypes across subjects. Therefore, we cannot conclude that different sport training modalities directly cause the measured index discrepancies. Future prospective longitudinal follow-up studies or controlled interventional trials are required to verify whether sport training can induce changes in body composition and vascular function.

## 5 Conclusion

In conclusion, athletes engaged in different sports display significant inter-group disparities in body composition and vascular function. Influenced by their sport-specific training, wrestlers tend to have relatively lower body fat levels, while tennis athletes present significantly higher FMD and lower CPP. All results only reflect cross-sectional associations between sport type and physiological indicators rather than training-derived causal changes. The correlational findings provide empirical reference for developing targeted exercise prescriptions aimed at body composition regulation and cardiovascular health maintenance.

## Data Availability

The original contributions presented in the study are included in the article/Supplementary Material, further inquiries can be directed to the corresponding author.
