# Supplementary material for: Sport-specific adaptations in body composition and vascular function: a comparative study of swimmers, tennis players, and wrestlers
Source: Front Sports Act Living. 2026 Jul 8;8:1832251. doi: 10.3389/fspor.2026.1832251 (PMC13388814; doi:10.3389/fspor.2026.1832251)
Supplement: Supplementary file 1 [file Table1.docx]

Table S1 Complete 24-outcome sensitivity summary

| Variable | Domain | ANOVA *F* | ANOVA *p* | *p* Bonf (K=24) | Bonf sig. | ANCOVA *F* group | ANCOVA *p* group | ANCOVA *F* sex | ANCOVA *p* sex | Max \|d\| | Pair |
| --- | --- | --- | --- | --- | --- | --- | --- | --- | --- | --- | --- |
| BMI | body comp | 2.47 | 0.0722 | 1.0000 | No | 2.78 | 0.0501 | 2.57 | 0.1148 | -0.98 | Tennis vs Control |
| PBF | body comp | 12.40 | <0.001 | 0.0001 | Yes | 14.11 | 0.0000 | 30.18 | 0.0000 | 2.60 | Swimming vs Wrestling |
| Fat mass | body comp | 22.21 | <0.001 | 0.0000 | Yes | 20.92 | 0.0000 | 5.87 | 0.0189 | -2.99 | Wrestling vs Control |
| Skeletal muscle | body comp | 5.33 | 0.0028 | 0.0665 | No | 5.53 | 0.0023 | 45.66 | 0.0000 | 1.30 | Wrestling vs Control |
| Body water | body comp | 4.74 | 0.0053 | 0.1268 | No | 6.61 | 0.0007 | 81.98 | 0.0000 | 1.31 | Wrestling vs Control |
| Protein | body comp | 4.41 | 0.0076 | 0.1833 | No | 6.07 | 0.0013 | 85.77 | 0.0000 | 1.28 | Wrestling vs Control |
| Minerals | body comp | 4.35 | 0.0082 | 0.1978 | No | 5.63 | 0.0020 | 59.14 | 0.0000 | 1.15 | Wrestling vs Control |
| baPWV L | arterial stiff | 0.34 | 0.7986 | 1.0000 | No | 0.48 | 0.6962 | 3.77 | 0.0577 | 0.46 | Tennis vs Wrestling |
| baPWV R | arterial stiff | 0.60 | 0.6153 | 1.0000 | No | 0.61 | 0.6135 | 2.23 | 0.1410 | 0.49 | Tennis vs Wrestling |
| cfPWV | arterial stiff | 3.74 | 0.0164 | 0.3944 | No | 3.78 | 0.0158 | 0.44 | 0.5085 | -1.35 | Tennis vs Control |
| ABI L | peripheral | 0.69 | 0.5613 | 1.0000 | No | 1.38 | 0.2579 | 9.04 | 0.0041 | -0.49 | Swimming vs Wrestling |
| ABI R | peripheral | 0.03 | 0.9938 | 1.0000 | No | 0.11 | 0.9539 | 3.58 | 0.0640 | 0.10 | Swimming vs Control |
| RestDiam | morphology | 0.65 | 0.5845 | 1.0000 | No | 0.47 | 0.7048 | 8.83 | 0.0045 | -0.54 | Tennis vs Wrestling |
| MaxDiam | morphology | 0.41 | 0.7474 | 1.0000 | No | 0.16 | 0.9214 | 7.36 | 0.0090 | 0.43 | Wrestling vs Control |
| FMD | endothelial | 7.73 | 0.0002 | 0.0054 | Yes | 8.29 | 0.0001 | 3.45 | 0.0687 | 1.41 | Tennis vs Wrestling |
| SBP | peripheral BP | 0.79 | 0.5035 | 1.0000 | No | 0.89 | 0.4517 | 12.78 | 0.0008 | 0.76 | Swimming vs Control |
| DBP | peripheral BP | 0.92 | 0.4391 | 1.0000 | No | 1.10 | 0.3595 | 4.27 | 0.0438 | 0.55 | Swimming vs Control |
| PP | peripheral BP | 0.46 | 0.7085 | 1.0000 | No | 0.34 | 0.7957 | 3.28 | 0.0759 | 0.33 | Swimming vs Tennis |
| CSBP | central BP | 5.69 | 0.0019 | 0.0450 | Yes | 7.47 | 0.0003 | 6.46 | 0.0140 | -1.78 | Wrestling vs Control |
| CDBP | central BP | 1.31 | 0.2818 | 1.0000 | No | 1.51 | 0.2221 | 3.31 | 0.0747 | 0.67 | Swimming vs Control |
| CPP | central BP | 12.08 | <0.001 | 0.0001 | Yes | 12.77 | 0.0000 | 1.66 | 0.2037 | -1.86 | Wrestling vs Control |
| AP | central BP | 1.25 | 0.2993 | 1.0000 | No | 0.81 | 0.4918 | 6.30 | 0.0152 | 0.72 | Wrestling vs Control |
| AIX@75 | wave reflection | 5.46 | 0.0024 | 0.0579 | No | 5.19 | 0.0033 | 0.14 | 0.7126 | -1.60 | Tennis vs Control |
| SEVR | cardiac perfusion | 5.82 | 0.0016 | 0.0393 | Yes | 6.00 | 0.0014 | 0.82 | 0.3682 | 1.59 | Swimming vs Control |

*Notes.* - Bonf sig. = Yes if the omnibus ANOVA *p* value survived Bonferroni correction with K = 24 (alpha’ = .05/24 = .00208). - ANCOVA: factor = sport group; covariate = sex (1 = male, 2 = female). - Max |d| = the largest absolute Cohen’s d among the six pairwise group contrasts, with the contributing pair shown in the final column. - All *p* values are two-tailed. *F* statistics from SPSS ONEWAY (ANOVA) and UNIANOVA (ANCOVA). - K = 24 simultaneous dependent variables for the family-wise correction.

Table S2 Sex-adjusted estimated marginal means and Bonferroni pairwise comparisons

*Note.* Estimated marginal means (EMMs) were calculated from ANCOVA models with sport group as the factor and sex as a covariate. Sex was evaluated at the sample mean (1.3684; sex coded 1 = male, 2 = female). Pairwise p values were Bonferroni-adjusted within each outcome across the six group contrasts.

Table S2A Estimated marginal means

| Outcome | Group | EMM | SE | 95% CI |
| --- | --- | --- | --- | --- |
| BMI | Swimming | 21.9 | 0.49 | [20.92, 22.88] |
| BMI | Tennis | 20.8 | 0.47 | [19.86, 21.74] |
| BMI | Wrestling | 21.86 | 0.44 | [20.97, 22.75] |
| BMI | Control | 22.72 | 0.47 | [21.77, 23.67] |
| Percent body fat | Swimming | 21.35 | 1.4 | [18.54, 24.17] |
| Percent body fat | Tennis | 19.19 | 1.35 | [16.48, 21.91] |
| Percent body fat | Wrestling | 11.62 | 1.28 | [9.06, 14.18] |
| Percent body fat | Control | 22.71 | 1.36 | [19.97, 25.44] |
| Fat mass | Swimming | 12.14 | 0.7 | [10.73, 13.55] |
| Fat mass | Tennis | 11.42 | 0.68 | [10.06, 12.78] |
| Fat mass | Wrestling | 7.8 | 0.64 | [6.51, 9.08] |
| Fat mass | Control | 15.22 | 0.68 | [13.84, 16.59] |
| Skeletal muscle | Swimming | 29.56 | 1.08 | [27.39, 31.72] |
| Skeletal muscle | Tennis | 30.57 | 1.04 | [28.49, 32.66] |
| Skeletal muscle | Wrestling | 32.11 | 0.98 | [30.15, 34.08] |
| Skeletal muscle | Control | 26.37 | 1.05 | [24.26, 28.47] |
| Body water | Swimming | 40.87 | 1.23 | [38.39, 43.34] |
| Body water | Tennis | 37.08 | 1.19 | [34.70, 39.46] |
| Body water | Wrestling | 41.35 | 1.12 | [39.10, 43.60] |
| Body water | Control | 35.01 | 1.2 | [32.60, 37.41] |
| Protein | Swimming | 11.04 | 0.34 | [10.35, 11.72] |
| Protein | Tennis | 10.06 | 0.33 | [9.40, 10.73] |
| Protein | Wrestling | 11.2 | 0.31 | [10.57, 11.82] |
| Protein | Control | 9.49 | 0.33 | [8.82, 10.16] |
| Minerals | Swimming | 3.98 | 0.12 | [3.73, 4.22] |
| Minerals | Tennis | 3.53 | 0.12 | [3.29, 3.76] |
| Minerals | Wrestling | 3.86 | 0.11 | [3.64, 4.08] |
| Minerals | Control | 3.38 | 0.12 | [3.14, 3.62] |
| FMD | Swimming | 9.72 | 0.57 | [8.57, 10.87] |
| FMD | Tennis | 12.39 | 0.55 | [11.28, 13.50] |
| FMD | Wrestling | 9.42 | 0.52 | [8.37, 10.47] |
| FMD | Control | 8.78 | 0.56 | [7.66, 9.90] |
| cfPWV | Swimming | 5.92 | 0.12 | [5.67, 6.18] |
| cfPWV | Tennis | 5.64 | 0.12 | [5.40, 5.89] |
| cfPWV | Wrestling | 6.02 | 0.11 | [5.79, 6.25] |
| cfPWV | Control | 6.21 | 0.12 | [5.96, 6.45] |
| Central systolic BP | Swimming | 102.63 | 2.09 | [98.44, 106.81] |
| Central systolic BP | Tennis | 98.58 | 2.01 | [94.55, 102.62] |
| Central systolic BP | Wrestling | 94.37 | 1.9 | [90.56, 98.17] |
| Central systolic BP | Control | 107.06 | 2.03 | [102.99, 111.13] |
| Central pulse pressure | Swimming | 30.49 | 1.39 | [27.70, 33.28] |
| Central pulse pressure | Tennis | 29.98 | 1.34 | [27.29, 32.67] |
| Central pulse pressure | Wrestling | 27.72 | 1.26 | [25.19, 30.26] |
| Central pulse pressure | Control | 38.67 | 1.35 | [35.95, 41.38] |
| AIx@75 | Swimming | 7.07 | 0.79 | [5.48, 8.67] |
| AIx@75 | Tennis | 7.22 | 0.77 | [5.68, 8.75] |
| AIx@75 | Wrestling | 8.35 | 0.72 | [6.90, 9.80] |
| AIx@75 | Control | 10.89 | 0.77 | [9.34, 12.44] |
| SEVR | Swimming | 206.74 | 7.01 | [192.67, 220.82] |
| SEVR | Tennis | 202.36 | 6.76 | [188.80, 215.92] |
| SEVR | Wrestling | 192.32 | 6.38 | [179.53, 205.12] |
| SEVR | Control | 169.16 | 6.82 | [155.47, 182.84] |

Table S2B Estimated marginal means

| Outcome | Contrast | Mean difference | SE | 95% CI | Bonferroni p |
| --- | --- | --- | --- | --- | --- |
| BMI | Swimming - Tennis | 1.1 | 0.68 | [-0.26, 2.46] | .664 |
| BMI | Swimming - Wrestling | 0.03 | 0.66 | [-1.29, 1.36] | 1.000 |
| BMI | Swimming - Control | -0.82 | 0.68 | [-2.18, 0.54] | 1.000 |
| BMI | Tennis - Wrestling | -1.06 | 0.65 | [-2.36, 0.23] | .631 |
| BMI | Tennis - Control | -1.92 | 0.67 | [-3.26, -0.58] | .035 |
| BMI | Wrestling - Control | -0.86 | 0.65 | [-2.17, 0.46] | 1.000 |
| Percent body fat | Swimming - Tennis | 2.16 | 1.95 | [-1.75, 6.07] | 1.000 |
| Percent body fat | Swimming - Wrestling | 9.73 | 1.9 | [5.92, 13.54] | < .001 |
| Percent body fat | Swimming - Control | -1.36 | 1.95 | [-5.28, 2.57] | 1.000 |
| Percent body fat | Tennis - Wrestling | 7.57 | 1.86 | [3.85, 11.30] | < .001 |
| Percent body fat | Tennis - Control | -3.51 | 1.92 | [-7.37, 0.35] | .441 |
| Percent body fat | Wrestling - Control | -11.09 | 1.88 | [-14.87, -7.30] | < .001 |
| Fat mass | Swimming - Tennis | 0.72 | 0.98 | [-1.24, 2.68] | 1.000 |
| Fat mass | Swimming - Wrestling | 4.34 | 0.95 | [2.43, 6.25] | < .001 |
| Fat mass | Swimming - Control | -3.08 | 0.98 | [-5.04, -1.11] | .017 |
| Fat mass | Tennis - Wrestling | 3.62 | 0.93 | [1.75, 5.49] | .002 |
| Fat mass | Tennis - Control | -3.8 | 0.96 | [-5.73, -1.86] | .001 |
| Fat mass | Wrestling - Control | -7.42 | 0.94 | [-9.32, -5.52] | < .001 |
| Skeletal muscle | Swimming - Tennis | -1.02 | 1.5 | [-4.02, 1.99] | 1.000 |
| Skeletal muscle | Swimming - Wrestling | -2.55 | 1.46 | [-5.48, 0.37] | .514 |
| Skeletal muscle | Swimming - Control | 3.19 | 1.5 | [0.18, 6.20] | .230 |
| Skeletal muscle | Tennis - Wrestling | -1.54 | 1.43 | [-4.40, 1.33] | 1.000 |
| Skeletal muscle | Tennis - Control | 4.21 | 1.48 | [1.25, 7.17] | .038 |
| Skeletal muscle | Wrestling - Control | 5.75 | 1.45 | [2.84, 8.65] | .001 |
| Body water | Swimming - Tennis | 3.79 | 1.71 | [0.35, 7.22] | .188 |
| Body water | Swimming - Wrestling | -0.48 | 1.67 | [-3.83, 2.86] | 1.000 |
| Body water | Swimming - Control | 5.86 | 1.72 | [2.41, 9.30] | .008 |
| Body water | Tennis - Wrestling | -4.27 | 1.63 | [-7.54, -1.00] | .070 |
| Body water | Tennis - Control | 2.07 | 1.69 | [-1.32, 5.46] | 1.000 |
| Body water | Wrestling - Control | 6.34 | 1.66 | [3.02, 9.66] | .002 |
| Protein | Swimming - Tennis | 0.97 | 0.47 | [0.02, 1.93] | .274 |
| Protein | Swimming - Wrestling | -0.16 | 0.46 | [-1.09, 0.77] | 1.000 |
| Protein | Swimming - Control | 1.54 | 0.48 | [0.59, 2.50] | .012 |
| Protein | Tennis - Wrestling | -1.13 | 0.45 | [-2.04, -0.22] | .093 |
| Protein | Tennis - Control | 0.57 | 0.47 | [-0.37, 1.51] | 1.000 |
| Protein | Wrestling - Control | 1.71 | 0.46 | [0.78, 2.63] | .003 |
| Minerals | Swimming - Tennis | 0.45 | 0.17 | [0.11, 0.79] | .060 |
| Minerals | Swimming - Wrestling | 0.11 | 0.16 | [-0.21, 0.44] | 1.000 |
| Minerals | Swimming - Control | 0.6 | 0.17 | [0.26, 0.94] | .005 |
| Minerals | Tennis - Wrestling | -0.33 | 0.16 | [-0.66, -0.01] | .250 |
| Minerals | Tennis - Control | 0.15 | 0.17 | [-0.18, 0.48] | 1.000 |
| Minerals | Wrestling - Control | 0.48 | 0.16 | [0.16, 0.81] | .027 |
| FMD | Swimming - Tennis | -2.67 | 0.8 | [-4.27, -1.07] | .009 |
| FMD | Swimming - Wrestling | 0.3 | 0.78 | [-1.26, 1.86] | 1.000 |
| FMD | Swimming - Control | 0.94 | 0.8 | [-0.67, 2.54] | 1.000 |
| FMD | Tennis - Wrestling | 2.97 | 0.76 | [1.44, 4.49] | .002 |
| FMD | Tennis - Control | 3.6 | 0.79 | [2.03, 5.18] | < .001 |
| FMD | Wrestling - Control | 0.64 | 0.77 | [-0.91, 2.18] | 1.000 |
| cfPWV | Swimming - Tennis | 0.28 | 0.17 | [-0.07, 0.63] | .674 |
| cfPWV | Swimming - Wrestling | -0.1 | 0.17 | [-0.43, 0.24] | 1.000 |
| cfPWV | Swimming - Control | -0.28 | 0.17 | [-0.63, 0.07] | .672 |
| cfPWV | Tennis - Wrestling | -0.38 | 0.17 | [-0.71, -0.04] | .165 |
| cfPWV | Tennis - Control | -0.56 | 0.17 | [-0.91, -0.22] | .011 |
| cfPWV | Wrestling - Control | -0.19 | 0.17 | [-0.52, 0.15] | 1.000 |
| Central systolic BP | Swimming - Tennis | 4.04 | 2.9 | [-1.77, 9.86] | 1.000 |
| Central systolic BP | Swimming - Wrestling | 8.26 | 2.82 | [2.59, 13.92] | .031 |
| Central systolic BP | Swimming - Control | -4.44 | 2.91 | [-10.27, 1.40] | .799 |
| Central systolic BP | Tennis - Wrestling | 4.22 | 2.76 | [-1.33, 9.76] | .798 |
| Central systolic BP | Tennis - Control | -8.48 | 2.86 | [-14.22, -2.74] | .027 |
| Central systolic BP | Wrestling - Control | -12.69 | 2.8 | [-18.32, -7.07] | < .001 |
| Central pulse pressure | Swimming - Tennis | 0.51 | 1.93 | [-3.36, 4.38] | 1.000 |
| Central pulse pressure | Swimming - Wrestling | 2.77 | 1.88 | [-1.00, 6.54] | .882 |
| Central pulse pressure | Swimming - Control | -8.18 | 1.94 | [-12.06, -4.29] | < .001 |
| Central pulse pressure | Tennis - Wrestling | 2.26 | 1.84 | [-1.43, 5.95] | 1.000 |
| Central pulse pressure | Tennis - Control | -8.69 | 1.9 | [-12.51, -4.87] | < .001 |
| Central pulse pressure | Wrestling - Control | -10.94 | 1.87 | [-14.69, -7.20] | < .001 |
| AIx@75 | Swimming - Tennis | -0.15 | 1.1 | [-2.36, 2.07] | 1.000 |
| AIx@75 | Swimming - Wrestling | -1.28 | 1.07 | [-3.43, 0.88] | 1.000 |
| AIx@75 | Swimming - Control | -3.82 | 1.11 | [-6.04, -1.60] | .007 |
| AIx@75 | Tennis - Wrestling | -1.13 | 1.05 | [-3.24, 0.98] | 1.000 |
| AIx@75 | Tennis - Control | -3.67 | 1.09 | [-5.86, -1.49] | .008 |
| AIx@75 | Wrestling - Control | -2.54 | 1.07 | [-4.68, -0.40] | .125 |
| SEVR | Swimming - Tennis | 4.38 | 9.74 | [-15.16, 23.93] | 1.000 |
| SEVR | Swimming - Wrestling | 14.42 | 9.49 | [-4.62, 33.46] | .808 |
| SEVR | Swimming - Control | 37.59 | 9.77 | [17.98, 57.20] | .002 |
| SEVR | Tennis - Wrestling | 10.04 | 9.28 | [-8.59, 28.67] | 1.000 |
| SEVR | Tennis - Control | 33.2 | 9.61 | [13.92, 52.49] | .007 |
| SEVR | Wrestling - Control | 23.17 | 9.42 | [4.26, 42.07] | .104 |

Table S3 Text-cited statistics highlighted in the Results

| Results location | Item | Statistic/value | Source |
| --- | --- | --- | --- |
| 3.1 Baseline characteristics | Sex distribution | Pearson chi2(3) = 2.03, p = .567 | Table 1; chi-square analysis |
| 3.1 Baseline characteristics | Sex distribution | LR G2 = 2.05, p = .562 | chi-square analysis |
| 3.1 Baseline characteristics | Sex distribution | Monte Carlo Fisher exact p = .584 | chi-square analysis |
| 3.2 Body composition | Wrestler fat mass | 7.59 ± 1.18 kg | descriptive statistics |
| 3.2 Body composition | Wrestler PBF | 10.70 ± 1.71% | descriptive statistics |
| 3.2 Body composition | Fat mass, Wrestling vs Control | Cohen's d = -2.99, 95% CI [-4.03, -1.95] | Cohen's d summary |
| 3.2 Body composition | PBF, Wrestling vs Control | Cohen's d = -2.10, 95% CI [-3.00, -1.21] | Cohen's d summary |
| 3.2 Body composition | Fat mass, Swimming vs Control | Cohen's d = -1.10 | Cohen's d summary |
| 3.2 Body composition | Fat mass, Tennis vs Control | Cohen's d = -1.19 | Cohen's d summary |
| 3.2 Body composition | Fat mass, Swimming vs Tennis | Cohen's d = +0.23 | Cohen's d summary |
| 3.3 Vascular function | FMD, Tennis vs Control | Cohen's d = +1.40, 95% CI [+0.57, +2.23] | Cohen's d summary |
| 3.3 Vascular function | FMD, Tennis vs Wrestling | Cohen's d = +1.41, 95% CI [+0.61, +2.21] | Cohen's d summary |
| 3.3 Vascular function | cfPWV, Tennis vs Control | Cohen's d = -1.35, 95% CI [-2.17, -0.53] | Cohen's d summary |
| 3.3 Vascular function | CPP, Swimming vs Control | Cohen's d = -1.29 | Cohen's d summary |
| 3.3 Vascular function | CPP, Tennis vs Control | Cohen's d = -1.43 | Cohen's d summary |
| 3.3 Vascular function | CPP, Wrestling vs Control | Cohen's d = -1.86 | Cohen's d summary |
| 3.3 Vascular function | CSBP, Wrestling vs Control | Cohen's d = -1.78 | Cohen's d summary |
| 3.3 Vascular function | SEVR, Swimming vs Control | Cohen's d = +1.59 | Cohen's d summary |
| 3.3 Vascular function | SEVR, Tennis vs Control | Cohen's d = +1.06 | Cohen's d summary |
| 3.3 Vascular function | SEVR, Wrestling vs Control | Cohen's d = +0.84 | Cohen's d summary |
| 3.3 Vascular function | AIx@75, Swimming vs Control | Cohen's d = -1.20 | Cohen's d summary |
| 3.3 Vascular function | AIx@75, Tennis vs Control | Cohen's d = -1.60 | Cohen's d summary |
| 3.3 Vascular function | AIx@75, Wrestling vs Control | Cohen's d = -0.99 | Cohen's d summary |
| 3.8 Sensitivity analyses | Bonferroni-significant outcomes | fat mass, PBF, FMD, CSBP, CPP, SEVR | Table 5; Supplementary Table S1 |
| 3.8 Sensitivity analyses | Left ABI sex covariate | F_sex = 9.04, p = .004 | Supplementary Table S1 |
| 3.8 Sensitivity analyses | Right ABI sex covariate | F_sex = 3.58, p = .064 | Supplementary Table S1 |
| 3.8 Sensitivity analyses | Mean ABI | F(3,53) = 0.22, p = .886 | mean ABI supplementary output |

*Note.* This table lists numerical results explicitly highlighted in the Results narrative. Values are reproduced from the analysis outputs, Cohen’s d tables, and supplementary ABI post-processing analysis. CI = confidence interval; LR = likelihood-ratio.

Table S4 Body Composition Indicators of Males and Females Across Groups

| Variable | Sex | Swimmers (n=13) | Tennis (n=14) | Wrestlers  (n=16) | Control (n=14) |
| --- | --- | --- | --- | --- | --- |
| Age (years) | M | 21.00±1.41 | 18.67±1.32 | 19.33±2.10 | 20.71±1.25 |
|  | F | 20.00±1.00 | 18.80±0.45 | 18.00±1.41 | 20.86±0.38 |
| Weight (kg) | M | 77.20±9.36 | 68.42±8.13 | 69.42±7.02 | 68.77±5.23 |
|  | F | 61.36±6.66 | 55.88±5.52 | 56.75±3.90 | 58.29±3.28 |
| Height (cm) | M | 182.00±6..95 | 173.55±6.19 | 176.58±4.27 | 173.93±4.03 |
|  | F | 169.00±4.95 | 163.00±5.43 | 164.00±4.24 | 158.57±5.06 |
| Body water (kg) | M | 46.30±5.33 | 40.78±2.67 | 44.45±6.39 | 40.04±3.78 |
|  | F | 31.70±2.77 | 30.78±2.39 | 37.38±2.19 | 27.01±3.27 |
| Protein (kg) | M | 12.56±1.49 | 11.13±0.68 | 12.06±1.78 | 10.94±1.03 |
|  | F | 8.46±0.73 | 8.24±0.73 | 10.13±0.56 | 7.20±0.87 |
| Inorganic Salts (kg) | M | 4.44±0.55 | 3.82±0.27 | 4.15±0.59 | 3.75±0.41 |
|  | F | 3.20±0.39 | 3.03±0.21 | 3.44±0.28 | 2.76±0.37 |
| Skeletal muscle (kg) | M | 33.06±3.90 | 31.59±2.03 | 34.45±5.32 | 30.96±3.09 |
|  | F | 23.64±2.27 | 28.98±3.93 | 28.58±1.74 | 19.84±2.70 |
| Body fat (kg) | M | 11.90±2.34 | 10.12±2.30 | 7.88±1.14 | 13.77±3.63 |
|  | F | 12.60±1.53 | 13.70±3.41 | 6.75±0.97 | 17.11±2.99 |
| BMI (kg/m²) | M | 22.40±2.00 | 20.70±1.73 | 22.24±1.71 | 23.11±2.64 |
|  | F | 21.06±1.57 | 21.00±1.61 | 21.10±0.50 | 22.11±1.18 |
| PBF (%) | M | 17.89±4.16 | 17.24±6.81 | 10.80±1.85 | 15.86±4.52 |
|  | F | 27.22±2.84 | 22.46±6.09 | 10.40±1.36 | 31.60±2.59 |

*Note.* M = Male, F = Female.

Table S5 Cardiovascular Function Indicators Between Males and Females Across Groups

| Variable | Sex | Swimmers (n=13) | Tennis (n=14) | Wrestlers  (n=16) | Control (n=14) |
| --- | --- | --- | --- | --- | --- |
| FMD (%) | M | 9.03±1.38 | 11.77±2.68 | 9.27±1.66 | 8.58±1.86 |
|  | F | 10.86±2.15 | 13.47±2.69 | 9.35±1.53 | 9.27±2.57 |
| SBP (mmHg) | M | 118.88±5.28 | 121.33±6.06 | 114.33±7.23 | 114.00±4.62 |
|  | F | 114.00±6.04 | 102.40±2.41 | 113.25±9.29 | 110.86±7.47 |
| DBP (mmHg) | M | 67.88±3.64 | 69.89±5.11 | 67.08±9.19 | 65.00±5.97 |
|  | F | 68.00±4.69 | 63.6±2.07 | 57.75±3.50 | 65.57±5.59 |
| PP (mmHg) | M | 51.00±3.25 | 51.44±5.76 | 47.25±5.15 | 49.00±4.86 |
|  | F | 46.00±4.64 | 38.8±2.95 | 55.50±7.94 | 45.29±10.48 |
| CSBP (mmHg) | M | 105.13±10.97 | 103.11±7.04 | 96.75±6.90 | 104.43±4.35 |
|  | F | 98.4±8.17 | 90.60±2.61 | 89.75±7.09 | 108.29±4.92 |
| CDBP (mmHg) | M | 73.13±8.97 | 70.67±6.02 | 68.50±10.00 | 68.14±3.13 |
|  | F | 70.40±5.94 | 65.00±2.35 | 62.75±6.13 | 67.71±5.38 |
| CPP (mmHg) | M | 32.00±4.04 | 32.44±2.24 | 28.25±3.96 | 36.29±5.09 |
|  | F | 28.00±4.74 | 25.60±1.52 | 27.00±2.16 | 40.57±8.96 |
| AIX@75 (%) | M | 7.00±3.70 | 7.00±2.18 | 8.25±3.02 | 10.86±2.54 |
|  | F | 7.20±4.44 | 7.60±2.19 | 8.50±2.08 | 11.00±2.65 |
| SEVR (%) | M | 203.00±14.37 | 194.22±31.46 | 186.67±23.26 | 181.43±35.26 |
|  | F | 213.00±9.14 | 216.80±29.58 | 206.25±9.60 | 158.57±18.08 |
| baPWV Left (m/s) | M | 9.42±0.79 | 9.72±0.68 | 9.38±0.87 | 9.93±1.50 |
|  | F | 9.48±0.74 | 9.35±0.61 | 8.88±0.40 | 8.83±1.10 |
| baPWV Right (m/s) | M | 9.46±0.62 | 9.86±0.79 | 9.44±0.71 | 9.54±1.14 |
|  | F | 9.37±0.90 | 9.40±0.75 | 9.08±0.36 | 9.06±1.28 |
| cfPWV (m/s) | M | 5.94±0.27 | 5.69±0.45 | 6.12±0.58 | 6.12±0.50 |
|  | F | 5.89±0.28 | 5.56±0.22 | 5.77±0.69 | 6.27±0.37 |
| ABI Left | M | 1.04±0.06 | 1.02±0.06 | 1.07±0.07 | 1.03±0.04 |
|  | F | 1.07±0.10 | 1.14±0.07 | 1.13±0.06 | 1.07±0.10 |
| ABI Right | M | 1.04±0.06 | 1.03±0.06 | 1.05±0.06 | 1.04±0.10 |
|  | F | 1.08±0.10 | 1.09±0.05 | 1.07±0.09 | 1.06±0.10 |

*Note.* M = Male, F = Female.
